# Man and His Enemies

**Published:** 1889-05-18

**Authors:** 


### THE DOCTOR'S PULPIT.

### MAN AND HIS ENEMIES.

### INVISIBLE COMPANIONS.

EVERYBODY can see a mountain! What microscopist can make visible a noxious smell? Yet the smell may be powerful enough to kill a king with fever, or to depopulate a country with pestilence. Modern life insists upon the use of brains; it insists as resolutely upon faith and works. "Look," it says, "look and see the circumstances in which you are placed. Hear what the sanitarian says to you, what the physiologist, what the physician says: hear and understand; believe and do." There is no widespread faith in the physical gospel which a true and admirable science has proclaimed; no general understanding and practice of its precepts. The ardent scientist desires to see the fruit of his labours. He and his fellows have made discoveries of infinitely more value to mankind than mountain ranges of gold mines or uncounted acres of diamond diggings. But those discoveries will take scores or even hundreds of years in penetrating the public mind; and some of them will never penetrate that mind at all. It is pitiful!

Why is a man not prosperous? Why is he not happy? Why is he not well? To a large extent because he has not had brains enough to solve the problems of his own life as they have presented themselves one by one to him. A problem came up at twenty which he failed to deal with wisely. He saw his mistake at thirty; but that was ten years too late. It is manifest, then, that a man may have a formidable enemy in his own brain. The understanding, however, is not always at fault. The inclination is the enemy which oftenest plunges us into mischief, and leaves us to get out of it as we can. A steamship is a noble object; but of what use is it without a helm? It must answer to the rudder, or it may run both captain, passengers, cargo, and itself upon rocks of the deadliest destruction. It is exactly so with a man. He must be guided by helm and rudder. If he were a lion or a wild bull, he might give way to every impulse of the moment without guilt, and with little danger. But he is not. He is a creature of intellect, who has an intellectual career before him—an intellectual destiny to work out—and Nature will not let him escape it.

But in insisting that the inclination shall be always obedient to the will and the will to the judgment, are we not presenting for acceptance a way of life which has the two fatal defects of being at once hateful and impossible? Even if we are, it is certain that science points with unswerving finger to that very way of life and to no other. No Sinaitic law is so stern and unbending as she. "Do this," she says, "and live"; "do that and die, and cover yourself with contempt." For science proclaims that at the present moment man has reached an unexampled height in pure intellect, that he has gained his unique eminence by the operation of natural forces, and that Nature insists upon demanding from him exactly that kind and degree of intellectual service she has for so many centuries been fitting him to perform. Nature is no soft and indulgent mistress who can be turned from her will by incapacity or persuasion. She is no creditor who can be paid in tears, or even in broken hearts. She is resistless as the sea and inflexible as death.

To the impulsive, the silly, the unreasoning, life is seldom worth living. If they happen to be rich, and to have some friend or relative who cares for them as for a costly toy or a pampered lap-dog, they may flutter on with a certain amount of inconsequent happiness, and die without ever having suspected that there were either duties or tragedies in the world. But that is the lot of few. The vast majority must learn to use judgment as the guide of will and the controller of inclination, or they will inevitably find the world a cage and a prison, full of disappointments and dangers, and destitute of every deep satisfaction and delight. Nature presents for acceptance a hard and difficult way of life. It may, indeed, be called, as the way of religion is called in the New Testament, a "narrow way." But it is the noble way of reason, of candour, of intelligence; the way from which prejudice is banished and stupidity is excluded; the way trodden by the feet of all who have separated themselves from vulgarity, wilfulness, mean selfishness, grossness of mind, and every form of baseness and mere animalism. It is the way, and the only way, worthy of intellectual beings; and every man and woman of any true worth strives more or less resolutely and steadily to walk in it.

But there are "invisible companions." If a Mephistopheles tempts every Faust to the ruin of his soul, as surely does a similar Mephistopheles tempt him to the ruin of his health or to the failure of his life purposes. If the external Mephistopheles can prevail nothing unless he finds in his Faust a willing and yielding mind, no more can the spirit of disease or of failure prevail unless he find some fatal defect of constitution or some weak mental disposition that yields to habits which contravene the laws of physiology. Mephistopheles, like every other external and invisible danger, is undoubtedly to be feared; but the unseen enemy most to be dreaded is that which has its home within the man himself—the enemy of prejudice, of intellectual idleness, of obstinacy, of self-complacency with things as they are. It is astonishing how each man and woman is so readily satisfied with his own knowledge and judgment of a thing. Every slut who can make a dumpling is ready at a moment's notice to take a cook's situation; and every post-office clerk fancies himself fit to be a bishop. The self-confident disposition is universal. It is as common in the capable man as in the fool. The docile temper of true science is as rare as the nightingale in a Northern clime. And yet the docile temper is the parent of all knowledge, of all safety, of all happiness, and of all permanent success.

The spirit of the learner is the spirit that casts out devils. All devils are dangerous, though they are not all equally dangerous. But the devils that lie in wait or that lurk within a man for the destruction of his physical health and life are not of a kind which he can afford to despise. No doctrine of science is more capable of proof than this: that life is a conflict—a struggle for survival. The corollary of the doctrine is equally evident and certain—viz., that the fittest are sure to survive. The true man hates defeat and failure, whatever shape they may assume. Not only does he hate them for himself, but he hates them for others. The pains, the struggles, the miseries, the heartbreaks, the defeats, the death of his fellow-men pierce his heart and write themselves with a pen of iron on his brain. If he knows, or thinks he knows, any panacea for any human ill, he cannot choose but persuade all men to believe in his gospel. A reasonable science is a panacea for many ills; a way of healthy life is a way which avoids many miserable pitfalls and passes through many pleasant countries. No persuasion can be too earnest, no arguments too high and sustained for the greatness of the subject. Health in its broadest sense is present and everlasting well-being. Health of body is one of the parents of health of mind, of character, and of fortune.

